# Improving health worker performance through text messaging: A mixed-methods evaluation of a pilot intervention designed to increase coverage of intermittent preventive treatment of malaria in pregnancy in West Nile, Uganda

**DOI:** 10.1371/journal.pone.0203554

**Published:** 2018-09-06

**Authors:** Christian Rassi, Georgia R. Gore-Langton, Badru Gidudu Walimbwa, Clare E. Strachan, Rebecca King, Sinwan Basharat, Celine Christiansen-Jucht, Kirstie Graham, Sam Siduda Gudoi

**Affiliations:** 1 Malaria Consortium, London, United Kingdom; 2 Malaria Consortium, Kampala, Uganda; 3 Department of Public Health and Policy, London School of Hygiene and Tropical Medicine, London, United Kingdom; 4 Cambridge Economic Policy Associates, London, United Kingdom; 5 The Nuffield Centre for International Health & Development, University of Leeds, Leeds, United Kingdom; Academic Medical Centre, NETHERLANDS

## Abstract

Poor health worker performance is a well-documented obstacle to quality service provision. Due to the increasingly widespread availability of mobile devices, mobile health (mHealth) has received growing attention as a service improvement tool. This pilot study explored feasibility, acceptability and outcomes of an mHealth intervention designed to increase coverage of intermittent preventive treatment of malaria in pregnancy (IPTp) in two districts of West Nile, Uganda. In both districts, selected health workers (N = 48) received classroom training on malaria in pregnancy. All health workers in one district (N = 49) subsequently received 24 text messages reinforcing the training content. The intervention was evaluated using a mixed-methods approach, including four focus group discussions with health workers and three in-depth interviews with district health officials, health worker knowledge assessments one month (N = 90) and six months (N = 89) after the classroom training, and calculation of IPTp coverage from participating health facilities’ (N = 16) antenatal care registers covering six months pre- and post-intervention. Complementing classroom training with text messaging was found to be a feasible, acceptable and inexpensive approach to improving health worker performance. The messages served as reminders to those who had attended the classroom training and helped spread information to those who had not. Health workers in the district where text messages were sent had significantly better knowledge of IPTp, achieving an increased composite knowledge score of 6.00 points (maximum score: 40) compared with those in the district where only classroom training was provided. Average facility coverage of three doses of IPTp was also significantly higher where text messages were sent (85.8%) compared with the district where only classroom training was provided (54.1%). This intervention shows promise for the improvement of health worker performance for delivery of IPTp, and could have significant broader application.

## Introduction

### Health worker performance and mobile health

Poor health worker performance has long been recognised as a major obstacle to quality health service provision, especially in low and middle income countries (LMICs) [1]. Interventions designed to increase health worker knowledge, skills and adherence to recommended clinical standards are typically job-related, support system-related or aimed at creating an enabling work environment [2], but the evidence base with regard to which interventions are most effective in resource-poor settings remains weak [3].

One approach that has received growing attention as a potential tool to improve health worker performance is mobile Health (mHealth), defined as the “use of mobile and wireless technologies to support the achievement of health objectives” [4]. Due to its potential to distribute information to large target audiences and at low cost over extended periods of time, even in hard-to-reach areas and among underserved populations, mHealth is increasingly seen as a scalable strategy for overcoming common health system constraints such as population growth, challenging access to services, inadequate workforce or limited financial resources [5]. The rapid expansion of mHealth in LMIC contexts over the last few years has been underpinned by the widespread availability of mobile phones [6] and people’s growing readiness to access health information through mobile devices [7]. In Uganda, almost 22 million mobile phones are registered in a population of 34.6 million. While this is below average in East Africa, the number of mobile phone subscriptions in Uganda is growing faster than in any other country in the region [8].

To date, mHealth approaches have most commonly been used in behaviour change interventions targeting patients, for example by sending text message reminders to improve adherence to chronic disease management guidelines [9]. More recently, mHealth interventions have occasionally targeted health workers, for example by facilitating emergency referrals, aiding data collection and reporting, strengthening communication with clients or the wider health system, and enhancing diagnosis and clinical decision making [10,11].

While enthusiasm for the use of mHealth solutions in resource-poor settings is strong, several challenges have been identified, including concerns over data security, a lack of standardisation and regulatory frameworks, and fragmented implementation in the absence of interoperable, open-access platforms [4]. It has also been pointed out that there is a lack of programmatic evidence to support scale-up of mHealth interventions in LMICs, with the vast majority of research describing pilot interventions and few well-designed studies providing robust evidence of impact at scale [12,13] or cost effectiveness [14,15]. Despite these limitations, there is broad agreement that mHealth has the potential to improve service delivery and health outcomes in general [9,14–16] and to boost health worker performance more specifically [10,11,17,18].

### Intermittent preventive treatment for malaria in pregnancy

Malaria infection during pregnancy can have harmful effects on mother and child. One of the recommended prevention and control mechanisms in areas of high and moderate malaria transmission in Africa is intermittent preventive treatment in pregnancy (IPTp), which entails administration of a curative dose of sulfadoxine-pyrimethamine (SP), an antimalarial drug, to all pregnant women, regardless of whether the individual is infected. IPTp is typically delivered to pregnant women as part of routine antenatal care (ANC).

At the time of the research (2014/15), the World Health Organization (WHO) recommended that pregnant women have at least four scheduled ANC visits by or under the supervision of a skilled attendant [19]. IPTp was recommended at each scheduled ANC visit, except during the first trimester and provided that doses were given one month apart [20]. When this study was conducted, Uganda had not yet adopted this most recent WHO IPTp policy recommendation. Reflecting earlier recommendations, national policies and guidelines at the time generally suggested that women should receive a maximum of two doses of SP.

While coverage of ANC is high in most African countries, uptake of IPTp has remained comparatively low, suggesting that opportunities for the provision of IPTp during ANC are being missed [21]. For example, 90% of women in Uganda attend ANC at least twice [22], while only 45% of women receive at least two doses of IPTp [23]. In 2013/14, Malaria Consortium conducted a qualitative study and a document and record review which explored barriers to IPTp uptake among women who attend ANC in Uganda. The study concluded that many missed opportunities for the provision of IPTp are due to inadequate health worker knowledge of the IPTp provision guidelines and poor service delivery practices, such as not advising women that it is safe to take SP on an empty stomach [24,25]. The results of this study informed the development of the pilot intervention that is presented in this paper.

## Materials and methods

### Study design

In collaboration with the Republic of Uganda’s Ministry of Health, Malaria Consortium developed and pilot tested an intervention that aimed to address health worker performance in terms of knowledge of IPTp, as well as adherence to national IPTp provision guidelines. Improved health worker performance was, in turn, expected to result in higher coverage of IPTp. The study was designed as a small-scale pilot, focusing on determining feasibility and acceptability of an intervention under programmatic conditions. The intervention consisted of two components:

- Provision of classroom training to health workers on malaria in pregnancy and IPTp
- Sending educational text messages after the training to reinforce the training content

The study also aimed to gather evidence of the intervention’s outcomes in terms of health worker knowledge and IPTp coverage, comparing classroom training plus text messaging with classroom training alone.

A convergent mixed-methods approach [26] was used to evaluate the intervention and arrive at an overall, descriptive assessment of its feasibility, acceptability and outcomes. This approach was considered appropriate in order to develop a rich, pragmatic and contextual understanding of how the intervention worked, allowing for an examination of both process and outcomes, and drawing on the strengths of different types of data [27]. Feasibility and acceptability were conceptualised using the UK Medical Research Council process evaluation framework for complex interventions [28], which distinguishes between fidelity, dose, adaptations, reach, participant responses and pathways to impact. The description of the text messaging component of the pilot intervention in this paper is informed by the mHealth evidence reporting and assessment (mERA) checklist [29]. Reporting of qualitative data is informed by the consolidated criteria for reporting qualitative research (COREQ) checklist [30].

### Study setting

**Fig 1:**
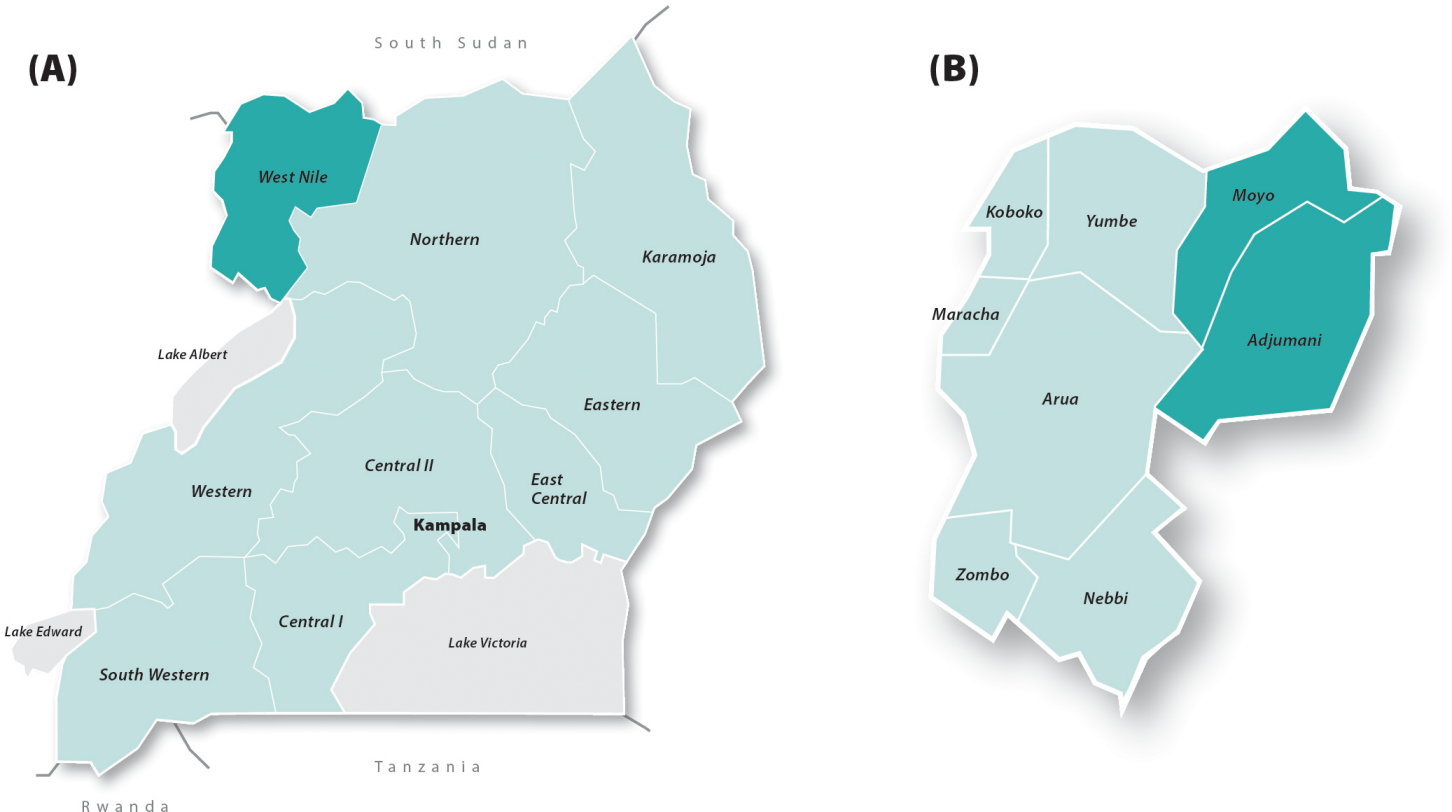
Maps of Uganda and West Nile. (A) Location of West Nile within Uganda highlighted dark green. (B) Location of study districts within West Nile highlighted dark green.

The study was conducted in two districts of West Nile: Moyo and Adjumani (Fig 1), with total populations of 139,000 and 225,000 respectively [31]. West Nile, one of the least developed regions of Uganda, was selected because it has historically been among the regions which have reported the lowest IPTp coverage in the country. According to the latest available household survey data, 35% of pregnant women received at least two doses of IPTp compared with the national average of 45% [23]. ANC attendance, on the other hand, is slightly above average, with 98% of mothers attending ANC at least once in West Nile, compared with the national average of 95% [22].

Moyo was selected purposively as it was one of the districts where Malaria Consortium had previously conducted research on barriers to IPTp uptake and it was therefore known that challenges relating to health worker performance affected IPTp provision in this district. Neighbouring Adjumani was selected as it was deemed similar to Moyo in terms of socio-demographic and geographical characteristics. Classroom training was provided in both study districts, but text messages were only sent to health workers in Moyo. Contamination between the two districts was expected to be minimal because the Nile serves as a physical barrier, with a small vehicle and passenger ferry as the only means of direct transport between the districts. Both districts are believed to be comparable to other remote districts in Uganda in terms of health workforce and service delivery challenges.

Taking into account limitations in terms of available time and budget, eight health facilities were selected in each study district, including all primary health care levels providing ANC according to national policy, as well as public and private not-for-profit facilities (Table 1). Private for-profit facilities were not included because they do not deliver ANC under the direct supervision of the Ministry of Health. The number of facilities selected for each facility type broadly corresponds to the share of this type of facility among all health facilities in the country [32]. Where more than the required number of facilities of a given type existed in the study districts, they were selected based on convenience in consultation with the District Health Offices in both study districts.

**Table 1:** Type and number of health facilities selected in each study district.

Health facility level	Public	Private not-for-profit
Hospital	1	0
Health Centre IV	1	0
Health Centre III	1	1
Health Centre II	3	1
**TOTAL**	**6**	**2**

### Intervention

#### Classroom training and training cascade

A training manual for a three-day health worker training on malaria in pregnancy was adapted by Malaria Consortium from a manual developed by the Ministry of Health’s National Malaria Control Programme, which was available in advanced draft form at the time. The training introduced updated IPTp provision guidelines in line with the most recent WHO policy recommendation of monthly IPTp administration after the first trimester.

In April 2015, six health workers (three female, three male) who had been identified as suitable trainers by the District Health Offices in the two study districts attended a three-day training of trainers, led by three master trainers from Malaria Consortium and the National Malaria Control Programme. In May 2015, the trainers conducted two three-day classroom trainings, one in each study district, which were attended by 48 health workers involved in the provision of ANC services at participating health facilities (Table 2). This represents approximately 50% of the health workforce for whose role the training content was relevant. Training participants had been selected by the District Health Offices in the two study districts in consultation with health facilities’ in-charges. At the end of the training, participants were tasked with sharing training information with colleagues involved in ANC who had not been selected to attend the training. This approach, often referred to as a training cascade, is a standard approach used for health worker training in many LMICs. As the study aimed to assess feasibility of the intervention under programmatic conditions, the study team did not intervene in the selection of health workers for classroom training attendance.

**Table 2:** Demographics of health workers who attended the classroom training (N = 48).

	n	%
**District**		
Adjumani	24	50.0
Moyo	24	50.0
**Sex**		
Female	38	79.2
Male	10	20.8
**Job category**		
Medical Officers	8	16.7
Nurses and Midwives	34	70.8
Nursing Assistants	6	12.5

N: number of health workers who attended the classroom training; n: number of health workers per socio-demographic category.

Following the training and with approval from the National Malaria Control Programme, health workers at the participating health facilities were expected to provide IPTp monthly after the first trimester ahead of the formal nationwide adoption of this policy. District health staff were expected to enforce the implementation of the new IPTp guidelines as part of their routine supervision activities. However, the study team did not intervene to support routine supervision.

#### Text messages

In collaboration with the National Malaria Control Programme and Reproductive Health Division, Malaria Consortium developed 24 text messages to reinforce the content of the malaria in pregnancy training, with a focus on the updated IPTp provision guidelines (S1 Table). Messages were in English, the language used for health worker education in Uganda, and observed a 160 character limit. They were pre-tested in two focus group discussions (FGDs) with a cohort of 17 health workers in another district of West Nile. Discussions focused on health workers’ understanding of the text messages, appropriateness to the context and determining the optimum time of the day for their dissemination.

All health workers providing ANC at the eight participating health facilities in Moyo district who had access to a mobile phone were targeted for the text messaging component. Provision of mobile phones was not part of the intervention. In consultation with the District Health Office and health facilities’ in-charges, 49 health workers were identified, including 22 of the 24 health workers who had attended the classroom training and the three trainers. Two health workers were lost to follow-up. Access to mobile phones was verified prior to the start of the intervention. All health workers identified indicated that they had access to a mobile phone and were hence enrolled in the text messaging component (Table 3).

**Table 3:** Demographics of health workers enrolled in the text messaging component (N = 49).

	n	%
**Sex**		
Female	39	79.6
Male	10	20.4
**Job category**		
Medical Officers	3	6.1
Nurses and Midwives	42	85.7
Nursing Assistants	4	8.2
**Attended classroom training**		
Yes	25	51.0
No	24	49.0

N: number of health workers enrolled in the text messaging component; n: number of health workers per socio-demographic category.

Text messaging started in June 2015, approximately one month after the completion of the classroom training. A different message was sent every weekday over a period of five weeks to participating health workers’ personal mobile phones. Messages were sent via mTrac, a short messaging service (SMS) platform designed to enable the Ministry of Health to communicate with care providers and to monitor health system performance based on real-time facility-level data. The system is integrated into the national District Health Information System (DHIS2) [33]. While mTrac is mainly used to allow care providers to send disease surveillance or stock supply data to a central server, it can also be used to send out bulk text messages from the central server to registered health care staff. At district level, the person tasked with maintaining the system and processing incoming data is typically a Biostatistician. For the pilot intervention, the Biostatistician at Moyo District Health Office was given a small allowance of 80,000 Ugandan shillings (approximately 20 United States dollars) for a mobile internet modem, which ensured access to the mTrac system remotely while travelling in areas with limited connectivity.

### Evaluation

#### Feasibility and acceptability

- Classroom training reports and participant feedback. At the end of the classroom training, health workers were asked to rate aspects of the training on a five-point scale with the help of a self-administered paper-based questionnaire (S1 File), which also contained an open-ended question prompting for any other comments. Data were entered into an Excel 2013 (Microsoft Corporation) spreadsheet and average scores were calculated for each item. Following each classroom training, the trainers submitted a report summarising agenda, objectives and challenges encountered, using a report template provided for this purpose. Trainers’ reports (S2 File) were reviewed and summarised by the study team.
- Text messages log. Sending of the messages was logged automatically by the mTrac system, but it was not possible to track whether messages had been received or opened. A copy of the mTrac log covering the days when text messages were sent (S1 Table) was kept and analysed.
- Supervision registers. In December 2015, participating health facilities’ supervision registers were reviewed for the six-month period following the classroom training. Supervision exchanges with District Health Office staff discussing malaria in pregnancy or IPTp were recorded in an Excel 2013 (Microsoft Corporation) spreadsheet (S2 Table) to assess if any improvements in knowledge or coverage could have been due to increased supervision levels.
- Pharmacy registers. The study team also reviewed participating health facilities’ pharmacy registers covering the six months post-classroom training and any stock-outs of SP were recorded in an Excel 2013 (Microsoft Corporation) spreadsheet (S2 Table) in order to assess the potential impact of periods of SP stock-outs on IPTp coverage.
- Context log. Throughout the study, the team kept a context log to record any external factors that could have affected implementation of the intervention or its outcomes.
- Focus group discussions and in-depth interviews. In each study district, two FGDs were conducted with health workers involved in the provision of ANC at the participating health facilities. FGD participants were purposively selected in consultation with the District Health Offices, ensuring representation from all participating health facilities, female and male health workers, a mix of different job titles and a combination of those who had and those who had not attended the classroom training. A total of 31 health workers participated in the FGDs (Table 4). In addition, three in-depth interviews (IDIs) were conducted with district health staff in the two study districts who were identified and selected by the study team based on their involvement in the intervention. This included the District Health Officers (one female, one male) in both study districts and the Biostatistician (male) in Moyo.

**Table 4:** Demographics of focus group discussion participants (N = 31).

	n	%
**District**		
Adjumani	15	48.4
Moyo	16	51.6
**Sex**		
Female	21	67.7
Male	10	32.3
**Job category**		
Medical Officers	3	9.7
Nurses and Midwives	24	77.4
Nursing Assistants	4	12.9
**Attended classroom training**		
Yes	17	54.8
No	14	45.2
**Received text messages**		
Yes	12	38.7
No	19	61.3

N: number of health workers who participated in focus group discussions; n: number of health worker per socio-demographic category.

Semi-structured discussion guides (S3 File) were developed by CR, RK, BG and GRGL. FGDs in both districts were used to explore participants’ views about the classroom training and whether information from the training had been shared with those who had not attended. In Moyo, FGDs additionally explored whether text messages had been received and read, participants’ opinions of the text messages and how they had affected health workers’ day-to-day job. IDI discussion guides explored respondents’ opinions of the intervention, contextual factors that may have affected intervention outcomes, as well as perceptions of the intervention’s scalability. As all discussion guides could only be used in a meaningful way with respondents who had been involved in the study, pre-testing the data collection tools was not feasible.

All qualitative data were collected in December 2015. FGDs and IDIs lasted between 60 and 90 minutes. They were conducted by GRGL in English with support from a female local research assistant who took notes during the FGDs. The research assistant received a one-day briefing from GRGL before the start of the field work. FGDs were audio-recorded and detailed reports were produced for each discussion by GRGL and the research assistant, summarising key points and including illustrative quotes, as well as the researchers’ observations. IDIs were audio-recorded and transcribed verbatim by GRGL.

Reports and transcripts were analysed thematically [34] using NVivo version 10 (QSR International) qualitative analysis software. All transcripts and reports were read by GRGL, RK and CR, who agreed an initial coding frame based on key themes identified. GRGL subsequently coded all data, discussing and agreeing changes to the coding frame with RK and CR based on themes emerging from close reading of the data (S3 Table). In a final step, GRGL summarised data by theme, highlighting differences between groups of respondents, as well as cross-cutting issues. Quality and consistency of the summaries was reviewed by RK.

#### Health worker knowledge

Health workers’ knowledge of IPTp was assessed using a self-administered, paper-based questionnaire (S4 File). The questionnaire consisted of ten multiple-choice questions, each with four possible answers of which one or more were true. All questions addressed issues covered during the classroom training. Six questions were specifically about the provision of IPTp, with the remaining questions relating to malaria in pregnancy more generally. The questionnaire was pre-tested with a cohort of 17 health workers from another district of West Nile to determine whether it was sufficiently sensitive to detect variation in knowledge. The same health workers participated in two FGDs to explore if instructions for completing the questionnaire were clear and questions were understood as intended.

The questionnaire was first administered at baseline, one month after the classroom training and just before the start of the text messaging in June 2015. The one-month gap between training and administering the questionnaire was considered sufficient to allow classroom training attendees to share training information with colleagues who had not been selected to attend. To test how knowledge had been shared and retained, the same questionnaire was administered at endline, in December 2015, six months after the classroom training and about four months after the last text message was sent.

At both baseline and endline, all health workers involved in the provision of ANC services at the participating health facilities in both study districts were targeted. Participants were identified in consultation with the District Health Offices and health facilities’ in-charges. The questionnaire was administered by members of the study team during visits to the participating health facilities at respondents’ usual place of work. A total of 90 and 89 health workers completed the knowledge assessment at baseline and endline respectively (Table 5).

**Table 5:** Demographics of health workers who completed the knowledge assessment at baseline (N = 90) and endline (N = 89).

	Baseline		Endline	
	n	%	N	%
**District**				
Adjumani	41	45.6	40	44.9
Moyo	49	54.4	49	55.1
**Sex**				
Female	72	80.0	72	80.9
Male	18	20.0	17	19.1
**Years in antenatal care**				
≤5	50	55.6	52	58.4
>5–10	16	17.8	14	15.7
>10	24	26.7	23	25.8

N: number of health workers who completed the knowledge assessment; n: number of health workers per socio-demographic category.

Of the 90 health workers who completed the knowledge assessment at baseline, 64 (71.1%) also completed it at endline. The remaining 26 health workers were lost to follow up due to staff turnover or absence, but replaced in almost equal numbers by health workers who were absent at baseline or joined a participating health facility at a later point. In Moyo, 40 of the 49 health workers who completed the endline questionnaire (81.6%) had received the text messages. **Table 6** shows levels of exposure to the intervention components among endline respondents.

**Table 6:** Exposure to intervention components among health workers who completed the endline assessment (N = 89).

	N	%
Attended classroom training and received text messages	18	20.2
Only received text messages	22	24.7
Only attended classroom training	12	13.5
Did not attend classroom training and did not receive text messages	37	41.6

N: Number of health workers who completed the endline knowledge assessment; n: number of health workers per level of exposure to intervention components.

Questionnaires were scored by awarding one point for each answer option correctly ticked as true or false and deducting one point for each answer option incorrectly ticked as true or false. Hence, the minimum score was -40, while the maximum score was 40. Data were double-entered into EpiData version 3.1 (EpiData Association) software. The two databases were then compared and, where differences between first and second entry were detected, data were verified against the paper questionnaire. STATA version 13.1 (StataCorp LLC) was used to calculate mean scores, 95% confidence intervals (CI) and p-values, as well as to perform regression. Scores were compared by regression between districts, sex, professional experience (defined as years of service in ANC), classroom training attendance and reception of text messages. Results were also compared between groups of participants with different levels of exposure to the intervention components. A difference-in-difference (DID) multivariate linear regression analysis using a random effects model was used to determine whether knowledge was significantly different between health workers in the two study districts over time. A further DID analysis was performed to adjust for factors that were found to be statistically associated with health worker knowledge.

#### IPTp coverage

A paper-based tool was used to record the following ANC and IPTp data from participating health facilities’ ANC registers, covering the six months before the classroom training (December 2014 to May 2015) and the six months following the classroom training (June 2015 to November 2015):

- Number of women who attended ANC for the first time (ANC1) during their most recent pregnancy
- Number of women who received the first, second, third and fourth (or higher) dose of IPTp (IPT1, IPT2, IPT3, IPT4+)

Data were extracted by members of the study team in December 2015 during visits to the 16 participating health facilities and subsequently entered into an Excel 2013 (Microsoft Corporation) spreadsheet (S2 Table). Monthly coverage was calculated using the following formula, where ‘n’ denotes the respective dose of IPTp:

$$IPTn\;coverage=\frac{Number\;of\;IPTn\;recorded}{Number\;of\;ANC1\;recorded}$$

Aggregate coverage for both districts was calculated for the six months prior to and post classroom training, which was considered the most informative cut-off point as it marked the introduction of the new IPTp provision guideline of monthly administration after the first trimester. Mean coverage was calculated by facility, using STATA version 13.1 (StataCorp LLC), and averages were compared to identify any differences between the two districts. A DID multivariate linear regression analysis using a random effects model was performed to compare the change in IPTp coverage between the two districts pre- and post-intervention. A further DID analysis was performed to adjust for any potential differences by facility.

### Timeline of intervention and data collection activities

**Fig 2:**
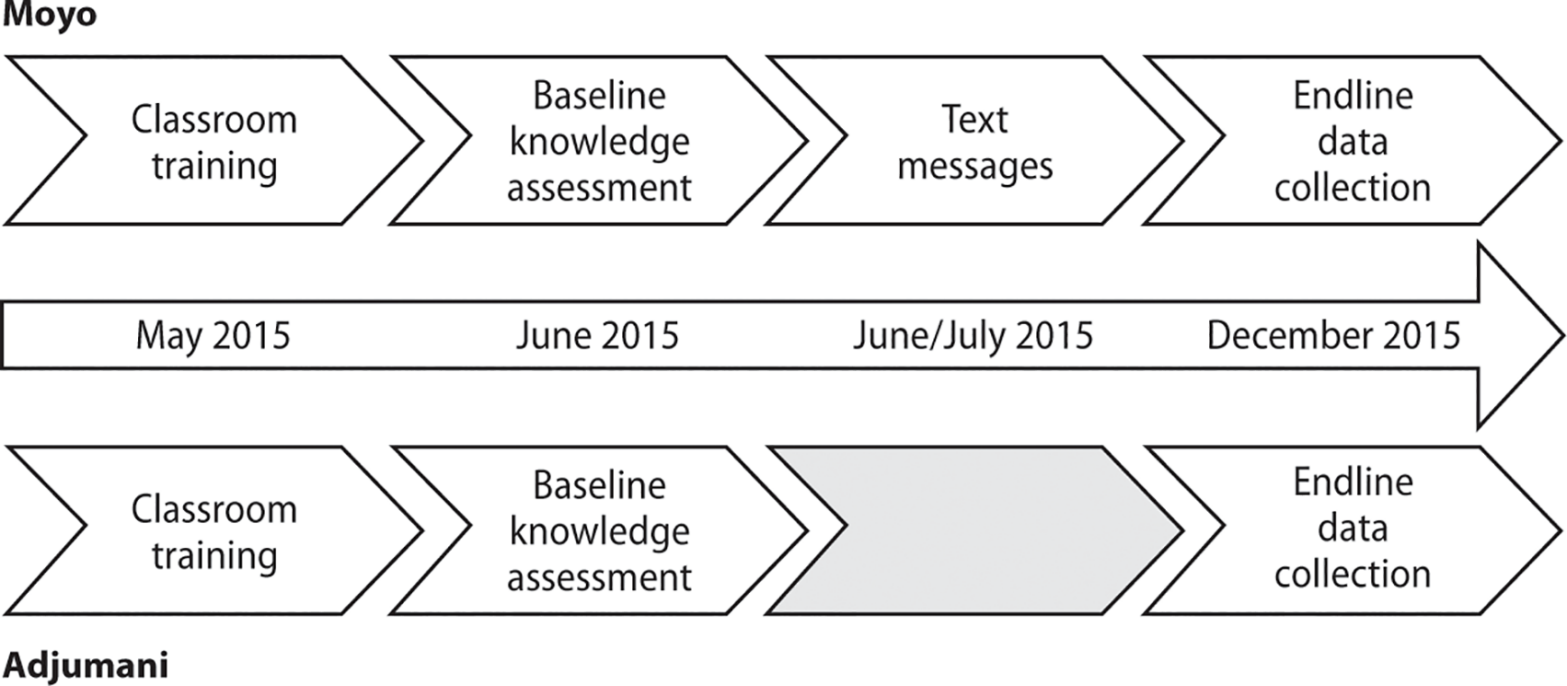
Timeline of intervention and evaluation activities in the two study districts. Endline data collection includes endline knowledge assessment, extraction of health facility data and focus group discussions/in-depth interviews.

Fig 2 shows the timeline of intervention and evaluation activities in the two study districts.

### Ethics

Ethical approval for the study, including the consent procedures used, was received from the School of Medicine Research Ethics Committee at the University of Leeds, UK (Reference: SoMREC/14/068), the Vector Control Division’s Ethical Committee at the Republic of Uganda’s Ministry of Health (Reference: VCD-IRC/047) and the Uganda National Council for Science and Technology (Reference: SS 3292). The classroom training was considered a routine Ministry of Health activity and consent for participation was therefore not taken. However, training attendees were informed that the training was provided as part of a research study. Written informed consent for receiving text messages and participating in knowledge assessments, FGD and IDIs was obtained from all participants with the help of standardised participant information sheets. None of the health workers who were approached refused to participate. As no data relating to individuals were collected as part of the extraction of health facility data, formal consent for this activity was not taken, though facility in-charges were informed of the research and gave oral consent for data extraction. All data were anonymised at the analysis stage. Mobile phone contact numbers of health workers in the intervention district were available in the mTrac system prior to the start of the intervention. While records for participating health workers were updated while obtaining consent and a recipient group was set up for the purpose of this study, this was considered a routine Ministry of Health activity. Therefore, standard Ministry of Health data security and privacy policies applied. No contact data was used for research purposes and no records containing contact data were kept by the study team.

## Results

### Feasibility and acceptability

#### Classroom training and training cascade

Trainers reported that the training agenda had been followed and training objectives had been met. Health workers’ satisfaction levels were generally very high. Overall quality, content and trainers’ performance were rated ‘good’ or ‘very good’ by all attendees. All responses to open-ended questions were positive, mostly commenting on the quality of the training and its value in improving service provision. Suggestions for improvement focused on enabling more frequent trainings and the inclusion of a wider number of health workers.

FGD participants who had attended the classroom training unanimously considered the training a positive experience. Many suggested that training on a variety of topics should be offered more frequently and that refresher or follow-up training would be beneficial. Health workers who did not attend the classroom training indicated that while they had been informed of the training, detailed information on the training contents had not been shared with them.

*Those who attended the training told them they attended the training about malaria in pregnancy, but the details about what they got from the training were not discussed. And they came with handouts which they requested we should read, but since we have laziness in reading, I don’t think some of us read it*.

FGD participant, Adjumani.

Effective training cascade mechanisms were a major concern for many FGD participants. In addition to more training opportunities, mentoring and support supervision were suggested in order to overcome this obstacle.

#### Text messages

All 24 text messages were sent out at approximately 10am each day on the day they had been scheduled. No system errors were logged during the implementation period. FGD participants from Moyo, where the text messages had been sent, generally stated that they had received and read the messages. Only a few participants reported that they had experienced problems. In a few cases, messages were received with a delay due to poor connectivity, sometimes at night. One health worker reported not receiving all the text messages due to network issues. A District Health Officer noted that the mTrac system could not send messages to users of one of the major mobile networks in Uganda, and that users of another, smaller network could only receive messages if they were in credit.

All FGD participants in Moyo indicated that they had appreciated receiving the text messages. No one reported that they had perceived the messages as disruptive or annoying. Participants who attended the classroom training agreed that the text messages helped them to remember what they had learned in the training.

*Some things you learn during the training, you can’t absorb everything in the brain. It’s a culture generally for them like when you come from the training, even if that package you got from the training is in the facility, you don’t have time to open, but that message you just open it and it will keep reminding you of what you have learnt from the training*.

FGD Participant, Moyo.

Several health workers reported sharing the information in the text messages with colleagues and friends.

*In a day the same message can be sent repeatedly and the following day another message so it will really give you that urge of reading, because it’s a different message not the old one. But before deleting, you first tell your friend about the message received and if they have also received it and if not, you share the information*.

FGD participant, Moyo.

The messages were also reported as having contributed to improving service delivery practices, including by participants who had not attended the classroom training. For example, it was pointed out that the messages may have served as reminders to encourage pregnant women who have concerns over taking IPTp on an empty stomach.

*In areas of malaria prevention in pregnancy, it helps to share with stubborn clients, because in their community they have people with different ideas. Like there are others who may wish not to take their drugs on DOT. The message can also help to him. It’s encouraging because it’s a guide, since one cannot remember everything. As you always move with the phone, it’s much easier to check. And in cases where a client is stubborn to adjust or are hard to change […] you can show them [the text message]*.

FGD participant, Moyo.

Most health workers were aware that the messages were sent through the mTrac system, as they recognised the sender’s number as the same number they use to text facility data to the District Health Office. The Biostatistician who had been tasked with sending out the text messages reported that setting up a user group on the mTrac system and entering the text messages into the system was simple, given that he was already familiar with the system. The daily task of sending the messages was reported to take just a few minutes and did not add significantly to the Biostatistician’s workload.

*It is part of what I do so I feel that I was doing my due responsibilities. So I feel it didn’t do any additions to this*.

Biostatistician, Moyo.

Both District Health Officers expressed support for the idea of using text messages to improve health worker performance.

*Yeah it is a good idea. In fact even for us here what we do it we have mTrac messages that we send to health centres. They normally read and respond and I think that is one of the good ideas and one of the practices we should encourage*.

District Health Officer, Moyo.

#### Health worker knowledge

At baseline, there was no statistically significant difference in knowledge scores between the two districts, with a mean score of 25.22 (95% CI: 22.86, 27.58) in Adjumani and 24.29 (95% CI: 21.78, 26.79) in Moyo. There was also no significant difference when health workers were separated by sex or professional experience. A significant difference (p<0.05) was only seen between health workers who had and those who had not attended the classroom training, with mean scores of 30.35 (95% CI: 27.99, 32.72) and 21.29 (95% CI: 19.42, 23.15) respectively.

At endline, the average score had decreased from baseline by 2.62 points to 22.60 (95% CI: 20.40–24.80) in Adjumani, where only classroom training had been provided, whereas in Moyo, where classroom training had been complemented by the text messages, it increased by 2.89 points to 27.18 (95% CI: 25.21, 29.16). The difference between the two districts at endline was statistically significant (p<0.001). There were also statistically significant differences between health workers who had attended the training and those who had not (p<0.001), as well as between those who had received the text messages and those who had not (p<0.001). Health workers with five to ten years of ANC provision experience had significantly worse knowledge scores than health workers with less experience (p = 0.002), although the small number of health workers in this category may not allow for a meaningful comparison (Table 7). Knowledge assessment results for each question have been provided as a supplementary file (S4 Table).

**Table 7:** Mean knowledge scores at endline (N = 89).

	n	Mean score (95% CI)	p-value^a^
**District**			
Adjumani	40	22.60 (20.40, 24.80)	
Moyo	49	27.18 (25.21, 29.16)	<0.001*
**Sex**			
Female	72	25.31 (23.56, 27.05)	
Male	17	24.35 (21.12, 27.59)	0.6
**Years in antenatal care**			
≤5	52	26.69 (24.90, 28.48)	
>5–10	14	19.42 (14.98, 23.88)	0.002*
>10	23	25.04 (24.96, 28.13)	0.25
**Attended classroom training**			
Yes	30	29.67 (27.21, 32.12)	
No	59	22.81 (21.14, 24.48)	<0.001*
**Received text messages**			
Yes	40	28.50 (26.46, 30.54)	
No	49	22.37 (20.44, 24.29)	<0.001*

N: number of health workers who completed the knowledge assessment at endline; n: number of health workers per socio-demographic category; CI: confidence interval.

^a^p-values relate to the difference in knowledge score within each category. For the “Years in ANC” category, they are based on the score among respondents with ≤5 years in ANC.

*indicates p value ≤0.05.

The DID between the two study districts was 5.52 points (95% CI: 2.67, 8.36) and statistically significant (<0.001). After adjusting for variables which were statistically associated with health worker knowledge, the DID increased to 6.00 points (95% CI: 3.52, 8.47), which was statistically significant (<0.001).

Analysing the data by level of exposure to the different intervention components revealed that health workers who did not attend the classroom training and did not receive the text messages had the poorest mean knowledge score at 21.08 (95% CI: 19.05, 23.11), compared with a much higher average score of 31.89 (95% CI: 29.30, 34.48) among health workers who had attended the training and received the text messages. Those who had received classroom training only and those who had received text messages only scored an average of 26.33 (95% CI: 21.74, 30.92) and 25.73 (95% CI: 23.07, 28.37) respectively.

#### IPTp coverage

**Fig 3:**
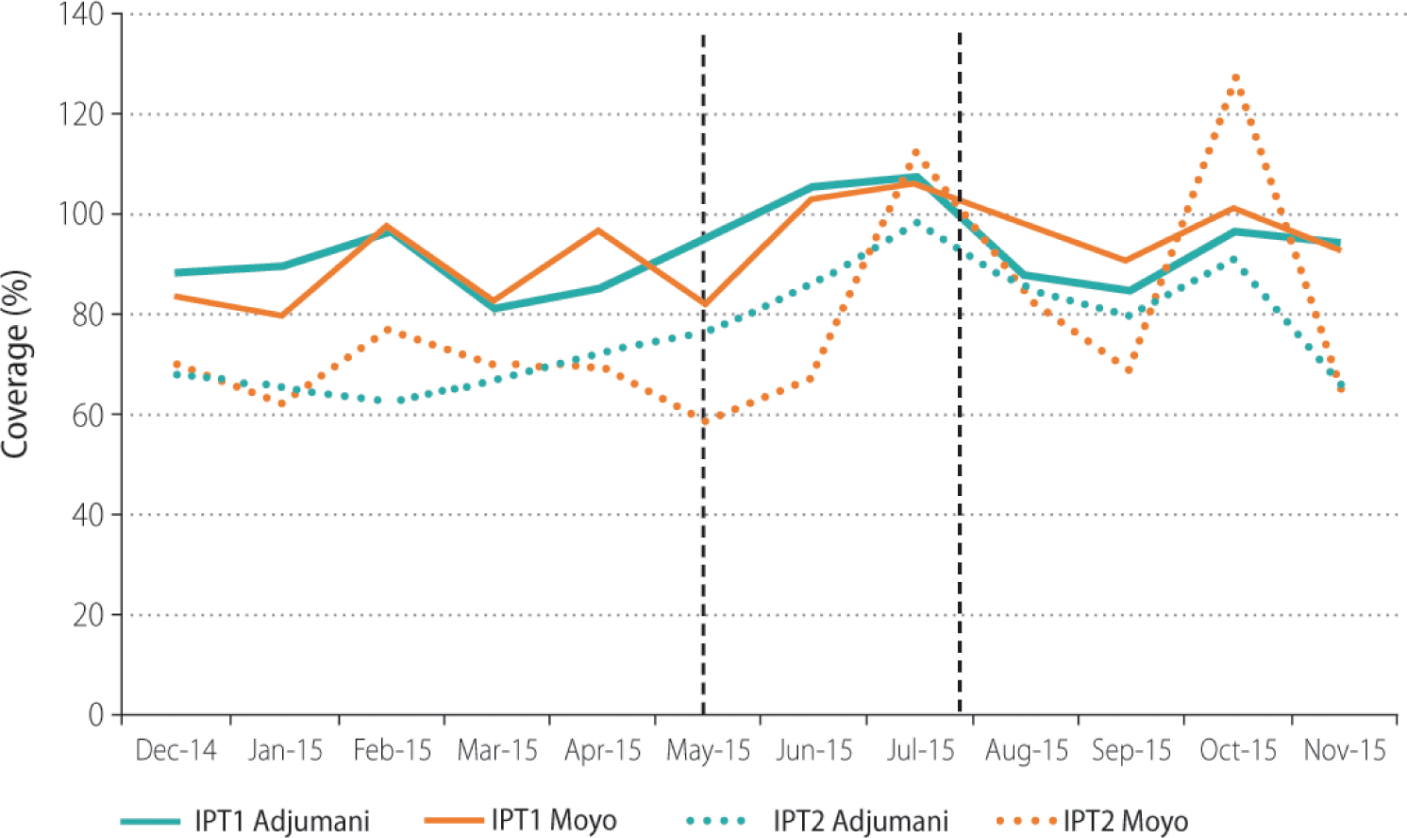
Monthly coverage of IPT1 and IPT2 at participating health facilities during six-month periods pre- and post-intervention in Adjumani and Moyo. The first vertical line marks the time of the classroom training (May 2015); the second vertical line marks the end of sending the text messages (July 2015). *IPT1*: *first dose of IPTp; IPT2*: *second dose of IPTp*.

**Fig 4:**
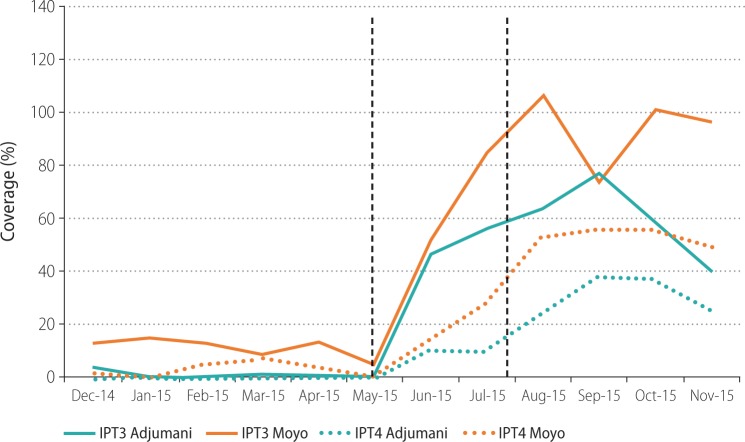
Monthly coverage of IPT3 and IPT4+ at participating health facilities during six-month periods pre- and post-intervention in Adjumani and Moyo. The first vertical line marks the time of the classroom training (May 2015); the second vertical line marks the end of sending the text messages (July 2015). *IPT3*: *third dose of IPTp; IPT4*: *fourth (or higher) dose of IPTp*.

Table 8 reports aggregate IPTp coverage in the participating health facilities for the six months before and after the classroom training by district and dose. Pre-intervention, coverage of IPT1 and IPT2 was high in both districts, while coverage of IPT3 and IPT4 low. Post-intervention, IPTp coverage increased in both study districts, especially for IPT3 and IPT4, with Moyo, where the text messages had been sent, consistently showing higher coverage than Adjumani, where only classroom training had been provided. Figs 3 and 4 illustrate monthly IPTp coverage in the two study districts pre- and post-intervention. Monthly coverage figures from all participating health facilities have been made available as a supplementary file (S2 Table).

**Table 8:** Aggregate IPTp coverage during six-month periods pre- and post-intervention at participating health facilities in Adjumani and Moyo.

	Pre-intervention coverage (%)		Post-intervention coverage (%)	
	Adjumani	Moyo	Adjumani	Moyo
**IPT1**	89.2	86.8	96.6	98.7
**IPT2**	68.8	67.6	84.1	84.8
**IPT3**	1.2	11.5	56.4	83.7
**IPT4+**	0.1	3.7	23.9	42.9

IPTp: intermittent preventive treatment for malaria in pregnancy; IPT1: first dose of IPTp; IPT2: second dose of IPTp; IPT3: third dose of IPTp; IPT4+: fourth (or higher) dose of IPTp.

For nominators and denominators, refer to supplementary file S2 Table.

Table 9 shows average facility coverage in the two districts by IPTp dose. There were no statistically significant differences pre-intervention. Post-intervention, there were no statistically significant differences for IPT1 and IPT2. However, coverage of IPT3 was significantly higher (p = 0.03) in Moyo (85.8%) compared to Adjumani (54.1%). While IPT4+ was also higher in Moyo (36.4%) than in Adjumani (20.6%), this was not statistically significant. The DID analysis showed similar results (Table 9). There were no significant differences for coverage of IPT1, IPT2 or IPT4 between the two districts. However, the DID between for IPT3 was 30.4% (95% CI: 0.2, 59.7) and statistically significant (p = 0.04). Adjusting for facility did not change this pattern.

**Table 9:** Average IPTp coverage during six-month periods pre- and post-intervention and difference-in-difference analysis.

	Pre-intervention coverage			Post-intervention coverage			DID (95% CI)	p-value^b^
	Adjumani (%)	Moyo (%)	Difference (p-value)^a^	Adjumani (%)	Moyo (%)	Difference (p-value)^a^		
**IPT1**	91.4	88.3	-3.1 (p = 0.6)	98.8	101.8	3.0 (p = 0.6)	6.2 (-10.2,-22.6)	0.4
**IPT2**	76.3	64.5	-11.8 (p = 0.3)	95.8	88.9	-6.9 (p = 0.5)	4.8 (-24.7, 34.4)	0.7
**IPT3**	4	5.3	1.3 (p = 0.8)	54.1	85.8	31.7 (p = 0.03*)	30.4 (1.6, 59.2)	0.04*
**IPT4+**	0.3	1.7	1.4 (p = 0.3)	20.6	36.4	15.8 (p = 0.1)	14.4 (-6.6, 35.4)	0.2

IPTp: intermittent preventive treatment for malaria in pregnancy; IPT1: first dose of IPTp; IPT2: second dose of IPTp; IPT3: third dose of IPTp; IPT4+: fourth (or higher) dose of IPTp; DID: difference-in-difference; CI: confidence interval.

^a^p-values relate to the difference in average coverage between the two districts for a given dose of IPTp.

^b^p-values relate to the difference-in-difference between the two districts for a given dose of IPTp.

*indicates p value ≤0.05.

## Discussion

Complementing conventional classroom training on malaria in pregnancy with sending educational text messages was found to be a feasible approach which was very well accepted by health workers and district health officials. There are also strong indications that the approach resulted in improved health worker performance in terms of knowledge of IPTp and increased coverage of IPTp. It is unlikely that outcomes were affected by increased routine supervision, prolonged periods of stock-outs or any other contextual factors external to the study. These findings are in line with those reported by two similar studies conducted in LMICs. In China, health workers’ knowledge of viral infections affecting the upper respiratory tract increased significantly after receiving text messages, compared with traditional continuing medical education [35]. In Kenya, sending text messages about malaria case management to health workers significantly increased correct management [36].

The intervention was generally delivered with high fidelity. The mTrac SMS platform was a suitable system for sending text messages to health workers. It was easy to use, inexpensive and reliable. Integration of the text messaging intervention with an existing platform was seen as a critical success factor by district health staff. This resonates with calls in the mHealth literature to avoid the creation of parallel systems and ensure competent use by district health staff tasked with maintaining the system without the need to invest in additional capacity building [12,14]. Other than a small allowance for mobile access to the system paid to the Biostatistician, no additional costs were incurred in setting up or sending the text messages. However, if the intervention were to be scaled up, there would most likely be costs associated with maintaining the mTrac system and ensuring an up-to-date database of health worker contact details.

Access to mobile phones and competent use of SMS technology was ubiquitous among study participants, which mirrors findings from several other studies in LMIC contexts [11,37]. While there are reports of technological or logistical barriers such as uncharged batteries or poor connectivity affecting mHealth interventions in the literature [10,14], in this study, occasional technical issues appeared to delay rather than preclude reception of the messages.

Health workers were unanimously supportive of the approach, which suggests that text messaging is seen as an adequate means of receiving educational information, in particular if the information comes from a trusted source and via a platform recipients are familiar with in a professional context. No concerns were raised over having work-related messages sent to personal phones. Health workers’ positive views of the intervention reflect the generally high acceptability of mHealth interventions among health workers reported by other studies [10,11,38]. While this may indicate that mobile phones are now seen as an integral tool that enables communication and information sharing in both personal and professional contexts, acceptability of frequent work-related text messages on personal phones will need to be monitored as mHealth applications targeting health workers multiply. There is also a risk of a growing taciturn expectation among policy makers and patients that health workers should bear the burden of mHealth–both morally in terms of accepting the blurring line between personal and professional lives and financially in terms of absorbing the costs of maintaining functioning mobile phones [39].

The most frequently mentioned benefit of the text messages was that they served as a reminder of classroom training content and good practices in service delivery, a benefit also identified in the Kenya study [38]. An important pathway by which the text messages complemented the classroom training appears to have been through strengthening the training cascade. Under programmatic conditions in LMIC settings, it is typically not practical to offer classroom training to all health workers for whose role the training would be relevant. However, tasking classroom training attendees with sharing information with colleagues who cannot attend does not on its own appear to be an effective approach, as relevant information does not reliably reach all those who would benefit. This was reported by FGD participants, but the limitations of the cascade approach are also illustrated by the significant gap in average knowledge score at baseline, one month after the classroom training, between those who had attended the classroom training and those who had not. The text messages, on the other hand, increased the reach of relevant information, including those who did not attend the classroom training because they were not selected or because they joined a participating health facility at a later stage. If mHealth interventions achieve impact through increasing access to and facilitating engagement with relevant information [40], text messaging health workers to remind them of service provision guidance may be effective because this approach gives a greater number of health workers access to relevant information. Text messages also appear to facilitate sharing and discussing of information among health workers, which may encourage behaviour change through shared action and the establishment of shared norms.

Both attendance at the classroom training and reception of the text messages were positively associated with health worker knowledge, which suggests that both components played a role in strengthening health workers’ capacity. There was a clear gradient of knowledge, with those who had not benefited from either of the two components exhibiting the lowest knowledge at endline, significantly outperformed by those who benefited from both. Those who only received one study component exhibited intermediary knowledge levels, with a slightly higher score among those who attended the training only compared with those who only received the text messages, albeit based on small samples. This gradient of knowledge supports the consensus in the literature that interventions aiming to improve health worker performance need to address a range of individual and systemic behaviour determinants. They are therefore more likely to succeed if they are multi-faceted [41–44], while simple interventions, such as providing classroom training or distributing written guidelines only, tend to be ineffective [1,3,41–43,45]. More research will be needed to determine the relative contribution of the two components. It was encouraging to see that the intervention resulted in positive outcomes in terms of health worker knowledge under programmatic conditions, in particular given the high staff turnover, which has been highlighted as a challenge in rural areas of Uganda [32]. The significant DID between health worker knowledge in the two districts illustrates that health workers in Moyo generally performed better over time, despite about one fifth of those assessed in this district at endline having joined a participating health facility after the text messages were sent. However, it should be noted that, while generally encouraging, knowledge scores at endline were still sub-optimal, with only those who benefited from both intervention components scoring more than 30 out of a possible 40 points. This suggests that further capacity building mechanisms, for example mentoring or support supervision, are required to achieve sustained quality of care, a conclusion echoed by the study conducted in China [35].

Improved knowledge of IPTp and the guidelines governing its provision through ANC appeared to lead to increased coverage of IPTp, with both intervention components likely to have played a role.

Coverage of IPT3 and IPT4 in particular increased in both study districts following the classroom-training, most likely due to a combination of two factors:

- IPT1 and IPT2 coverage was high compared with the national and regional average across all participating health facilities during the six months prior to the intervention and there was therefore little room for improving coverage rates further.
- The main change in practice necessitated by the updated IPTp provision guidelines relates to the provision of more than two doses of IPTp, which was not required under previous guidelines. Consequently, coverage of IPT3 and IPT4 was very low pre-training and increased substantially once the new guidelines had been communicated.

The significant DID between the two districts pre- and post-intervention for IPT3 suggests that the text messages contributed to better performance in terms of adherence to provision guidelines. Note that since this study was conducted, WHO has issued new recommendations for the provision of ANC, which now call for eight contacts between the mother and a healthcare provider [46]. IPTp should be provided as early as possible during the second trimester and at each subsequent contact with a health worker, ensuring that doses are administered one month apart [47]. Classroom training and text messages will need to be adapted accordingly if used in future interventions.

Number, frequency and duration of the text messages in this intervention (24 messages, one individual message per week day, five weeks) were comparable to the study conducted in China (16 messages, three messages per week, duration not specified) [35]. The study conducted in Kenya, however, adopted a different approach, with only ten individual messages sent out at a rate of two messages per day over six months [36]. Taken together, findings from the three studies involving sending educational text messages to health workers suggest that text messaging, at a relatively low dose, can improve health worker performance and that text messages do not need to be repeated over an extended period of time. Providing additional motivation, for example inspirational quotes included in messages in the Kenya study [38], does also not seem to be required.

### Limitations

A major limitation of the study is its pilot study design, including lack of randomisation and small sample size, which means it is not possible to conclusively attribute outcomes in terms of health worker knowledge and IPTp coverage to the intervention or to assess the degree to which different intervention components contributed to its outcomes. The study does therefore not answer the frequently cited call for robust evidence of the impact of mHealth interventions implemented at scale. Similarly, the study does not provide evidence of the text messaging component’s cost effectiveness. However, the role of well-designed pilot studies in conceptually demonstrating how health system constraints can be addressed is widely acknowledged [48,49]. This study provides robust evidence of feasibility and acceptability of an intervention under programmatic conditions, including an innovative mHealth component, and goes beyond most small-scale studies by systematically gathering evidence of the intervention’s potential outcomes, as well as potential confounding factors.

Another limitation of the study is that its design was not based on behaviour change theory. Study results have been reviewed and discussed in light of theoretical considerations, but this was a post hoc process. The development of the intervention was, however, informed by rigorous formative research, which has been highlighted as a crucial success factor for public health interventions [50].

The study could have been strengthened by a component exploring health worker skills and adoption of good service delivery practices as necessary intermediary steps between improved knowledge and increased IPTp coverage. However, given limitations with regard to available time and funding, this was not feasible and the link between increased knowledge and increased coverage has to be inferred.

Coverage rates extracted from facility registers may be unreliable as poor recording practices have been described in the literature [24,51] and observed in this study. Moreover, the formula used to calculate IPTp coverage, which uses ANC1 as the denominator, necessarily results in a crude measure of coverage. It would be more precise to use the number of all women visiting ANC and eligible for a given dose as the denominator. However, facility register data are still likely to be the most accurate readily available data source and using ANC1 as the denominator for calculating IPTp coverage is common practice [52].

Finally, the social desirability bias inherent in all qualitative work and the potential presence of a Hawthorne effect should be acknowledged. However, best practice with regard to conducting qualitative research was adopted and no evidence was found that awareness of participating in the study may have affected study results.

## Conclusions

It has been suggested that in-depth understanding of mHealth interventions requires multidimensional, mixed-methods evaluation approaches [11]. This study applied a robust mixed-methods design to assess feasibility, acceptability and determine potential outcomes of an intervention which involved combining conventional classroom training with sending educational text messages to health workers. Findings suggest that this approach is feasible and acceptable in the context of malaria in pregnancy and IPTp. The approach has the potential to improve health worker knowledge and coverage of IPTp. Despite the growing body of evidence on mHealth in general, this study adds to the comparatively small number of examples describing an mHealth intervention that addresses health service provider training and education [48]. It indicates that text messaging, in combination with other capacity building tools and approaches, is a promising approach for achieving behaviour change among health workers. To conclusively assess impact at scale, more research needs to be conducted. Results and insights from this pilot study could inform the design of a future trial, including estimating effect size to underpin sample size calculations, recruitment and consent procedures, and data collection tools [49].

Combining classroom training with text messaging may be an approach that is applicable to improving health worker performance beyond malaria in pregnancy, especially where text messaging platforms already exist, the desired behaviour change is comparatively simple and health worker performance has been determined as a key obstacle to quality of care. If scaled-up and applied to other training topics, a strategic and coordinated approach will be required in order to ensure consistency of messaging and avoid text messaging fatigue among health workers. Similarly, while text messaging platforms typically offer two-way communication functionality and interactivity has been described as a potential success factor of mHealth applications [17], introducing an interactive element to this intervention would require significantly increased effort and investment, and the appeal of the approach rests, to a large extent, in its simplicity.

## Supporting information

S1 Table Text messages and text messages log. IPTp: intermittent preventive treatment for malaria in pregnancy; ANC: antenatal care; SP: sulfadoxine-pyrimethamine; DOT: directly observed treatment; HIV: Human Immunodeficiency Virus.
(PDF)

S2 Table Antenatal care, intermittent preventive treatment in pregnancy, stock-out and supervision data. ANC: antenatal care; ANC1: first ANC visit; IPTp: intermittent preventive treatment for malaria in pregnancy; IPT1: first dose of IPTp; IPT2: second dose of IPTp; IPT3: third dose of IPTp; IPT4+: fourth (or higher) dose of IPTp.
(XLSX)

S3 Table Coding frame. IPTp: intermittent preventive treatment for malaria in pregnancy; SP: sulfadoxine-pyrimethamine; CME: continuing medical education; ANC: antenatal care; mHealth: mobile health.
(PDF)

S4 Table Knowledge assessment scores by question. *Difference-in-difference adjusted for clustering by facility, facility type, training attendance and years of service in antenatal care.
(XLSX)

S1 File Classroom training participants’ evaluation questionnaire. (PDF)

S2 File Trainers’ training reports. (PDF)

S3 File Focus group and in-depth interview discussion guides. (PDF)

S4 File Knowledge questionnaire. (PDF)
