# Supplementary material for: Improving health worker performance through text messaging: A mixed-methods evaluation of a pilot intervention designed to increase coverage of intermittent preventive treatment of malaria in pregnancy in West Nile, Uganda
Source: PLoS One. 2018 Sep 6;13(9):e0203554. doi: 10.1371/journal.pone.0203554 (PMC6126848; doi:10.1371/journal.pone.0203554)
Supplement: S1 Table — IPTp: intermittent preventive treatment for malaria in pregnancy; ANC: antenatal care; SP: sulfadoxine-pyrimethamine; DOT: directly observed treatment; HIV: Human Immunodeficiency Virus. (PDF) [file pone.0203554.s001.pdf]

Text messages and text messages log

Date sent	Text message	System errors
29/06/15	Over the next few weeks, you will receive messages about the dangers of malaria in pregnancy and the importance of providing IPTp to women attending ANC.	None
29/06/15	You will receive about 20 messages in total. The first message will be sent on Tuesday, 30 June, from this number: 6767. Please share with colleagues.	None
30/06/15	When a pregnant woman has malaria, it is dangerous for the mother and her unborn child. In the most severe case, both mother and baby can die.	None
1/07/15	Pregnant women are more susceptible to malaria because their immunity is low. Malaria in pregnancy will cause severe anaemia in many pregnant women.	None
2/07/15	Malaria in pregnancy is a leading cause of spontaneous abortion. If the baby survives, it may often be weak and more likely to get sick.	None
3/07/15	The malaria parasite will often hide in a pregnant woman's placenta and may not be detectable in her bloodstream.	None
6/07/15	Even pregnant women who look and feel well may have malaria. This is why all women should receive medication to protect them from the disease.	None

07/07/15	Pregnant women should receive a drug called SP monthly beginning in the second trimester to prevent adverse consequences of malaria. This is called IPTp.	None
08/07/15	The first dose of SP should be given as early as possible during the second trimester. SP is safe until the time of delivery.	None
09/07/15	More doses of IPTp increase women's protection from malaria. IPTp should be given repeatedly as long as there are 4 weeks between doses.	None
10/07/15	You should give IPTp whenever a woman attends ANC after the first trimester until delivery, as long as there are at least 4 weeks between doses.	None
13/07/15	IPTp should always be taken at the health facility under the supervision of a health worker (DOT).	None
14/07/15	SP is safe to take on an empty stomach and women should be encouraged to take IPTp at the facility regardless of whether or not they have eaten.	None
15/07/15	SP works less well these days as malaria treatment, but is still very effective at reducing the number of malaria parasites in the placenta.	None
16/07/15	Sometimes, women may experience mild side effects like nausea or dizziness after taking SP, but they should still be encouraged to take the medication.	None

17/07/15	Only those who experienced severe side effects such as a rash or difficulties breathing after taking IPTp should not receive SP. Such instances are rare.	None
20/07/15	Pregnant women who are taking co-trimoxazole or other sulpha-containing drugs should not be given IPTp. Many HIV positive women take co-trimoxazole.	None
21/07/15	Most women trust health workers. When providing IPTp, tell women why IPTp is important and that it is safe. Encourage them to take the medication as DOT.	None
22/07/15	Remind women attending ANC that for added protection from malaria in pregnancy, they should also sleep under an insecticide treated mosquito net.	None
23/07/15	Also remind pregnant women attending ANC to visit a health centre immediately if they have any signs of malaria to test and receive treatment.	None
24/07/15	Remember: give IPTp whenever a woman attends ANC after the first trimester until delivery, as long as there are at least 4 weeks between doses.	None
27/07/15	There is no danger in taking SP on an empty stomach. Mild side effects are possible, but are outweighed by the benefit of a healthier pregnancy.	None
28/07/15	Remember to record every dose of SP you provide in the ANC register and on the ANC card and the mother's ANC card.	None

29/07/15	This is the last message about malaria in pregnancy and IPTp. If you have any questions, don't hesitate to ask your in-charge or district health team.	None
