# Supplementary material for: Improving health worker performance through text messaging: A mixed-methods evaluation of a pilot intervention designed to increase coverage of intermittent preventive treatment of malaria in pregnancy in West Nile, Uganda
Source: PLoS One. 2018 Sep 6;13(9):e0203554. doi: 10.1371/journal.pone.0203554 (PMC6126848; doi:10.1371/journal.pone.0203554)
Supplement: S3 Table — IPTp: intermittent preventive treatment for malaria in pregnancy; SP: sulfadoxine-pyrimethamine; CME: continuing medical education; ANC: antenatal care; mHealth: mobile health. (PDF) [file pone.0203554.s003.pdf]

Coding frame

Conceptual framework	Themes	Sub-themes
Classroom training	Knowledge gained	Malaria in pregnancy
		Change in IPTp guidelines
		Content of IPTp guidelines
		Provision practices
		Contraindications
		Adverse effects of SP
		Data recording
	Attendees	Selection of health workers to attend
		Number of staff to attend
	Format and logistics	Frequency of trainings
		Topics of trainings
		Attendee expenses
		Cost of training
	Opinions	Structure
		Clarity
		Content
		General opinions
Post-training	On-the-job training	Frequency
		Format
	Follow-up classroom training	Demand

		Frequency
	Changes in practice	Malaria in pregnancy management
		Provision of IPTp
		Recording practices
		Information sharing
Information cascade	Barriers	Attitudes
		Staff turnover
		Time constraints
		CME
		Other
	Facilitators	How information is shared
		What information is shared
		When information is shared
		Who information is shared with
	Mechanisms	CME
		Text messages
		Other
	Opinions	Cascade approach
		Other
Text messages	Content	Text messages
	Purpose	Reminder

		Information sharing
		Engagement
		Teaching new staff
	Format	Timing
		Language
	Delivery	Internet
		mTrac
		Network
		Updating contact details
		Setting up messages
		Sending messages
		Costs
		Problems
	Scalability	Roles and responsibilities
		Other topics
		Other audiences
		Other geographies
Training plus text messages	Combined effect	Value added
		Complementarity
Data recording	Tools	ANC register
		ANC cards
	Job aid	Availability
		Use

		Non-use
		Opinions
		Suggestions
Context	Population	Migration and changes
		Conflict
	Climate	Rain
	Other programmes	mHealth interventions
		Health service delivery interventions
		Implementing partners
Health system	Supervision	Frequency
		Format
		Topics
		Barriers
	Stock-outs	Stock levels
		Redistribution
		Supply chain
Malaria Consortium	Text messaging intervention	Awareness
		Information
		Purpose
	General	Comments on work
		Requests for support
