# Supplementary material for: Improving health worker performance through text messaging: A mixed-methods evaluation of a pilot intervention designed to increase coverage of intermittent preventive treatment of malaria in pregnancy in West Nile, Uganda
Source: PLoS One. 2018 Sep 6;13(9):e0203554. doi: 10.1371/journal.pone.0203554 (PMC6126848; doi:10.1371/journal.pone.0203554)
Supplement: S1 File — (PDF) [file pone.0203554.s005.pdf]

Training participants' evaluation questionnaire

Dates of the training ________________________________

District in which training done ______________________________

Please rate the below aspects of the training by ticking one of the boxes from Excellent (5) to Very poor (1). This questionnaire is completely anonymous.

	Excellent	Good	Neutral	Poor	Very poor
	5	4	3	2	1
The overall quality of the training					
The content of the training					
The way in which the trainer led the training sessions and how they taught the information					
Availability of the materials needed for the training (stationary, manuals etc.)					
The venue for the training sessions (its appropriateness for the training and the number of participants)					
The food and refreshments provided					

Please leave any other general comments or thoughts

____

____

____

____
