# Supplementary material for: Improving health worker performance through text messaging: A mixed-methods evaluation of a pilot intervention designed to increase coverage of intermittent preventive treatment of malaria in pregnancy in West Nile, Uganda
Source: PLoS One. 2018 Sep 6;13(9):e0203554. doi: 10.1371/journal.pone.0203554 (PMC6126848; doi:10.1371/journal.pone.0203554)
Supplement: S2 File — (PDF) [file pone.0203554.s006.pdf]

Training report – Adjumani

DATE: 27th to 29th MAY 2015

EXECUTIVE SUMMARY

This report is about what transpired during the Malaria in Pregnancy training for front line health workers in Adjumani District .This took place at Anne's guest house between 27th and 29th May 2015.

The training was planned to last for 3 days and it was organized for health workers who take care of pregnant women and provide other ANC services in the facilities.

A total of 24 (six males, eighteen females) participants were identified to take part in this training and all of them attended.

Participants included, (see table below):

	**Adjumani**		
**Cadre**	**Female**	**Male**	**TOTAL**
Clinical Officers	00	01	01
Nursing Officers	04	01	05
Enrolled Comprehensive Nurses	03	02	05
Enrolled Midwives	11	00	11
Enrolled Nurses	00	02	02
**TOTAL**	**18**	**06**	**24**

Background

The World Health Organisation (WHO) recommends intermittent preventive treatment (IPTp) with Sulfadoxine-Pyrimethamine (SP) for the prevention and control of malaria in pregnancy in all areas with moderate to high malaria transmission in Africa. The government of Uganda has set a target of 85 percent of pregnant women receiving at least two doses of IPTp (IPT2) delivered as part of the focused antenatal care (ANC) package. However, despite high ANC attendance and increased efforts to improve IPT2 coverage by government and implementation partners, this target is far from being met.

The ministry of health recognises some of the challenges associated with IPTp uptake Uganda such as; lack of knowledge about IPTp policy guidelines among health workers, poor data recording at health facility level and inadequate support supervision.

The purpose of this training therefore, was to update health workers knowledge about IPTp and improve the health management information system through training of frontline health service providers, particularly ANC staff.

The training emphasized the provision of improved ANC services as a way of increasing uptake of especially Malaria in Pregnancy prevention services.

Objectives

1. To improve health workers knowledge in management of malaria in pregnancy through updating them about the MIP policy
2. To orient Health Workers on records management including the job aid for MIP

Content

1. Malaria in pregnancy- effects on the mother and baby
2. Review of goal oriented ANC
3. National Malaria in Pregnancy policy and strategy
4. Management of malaria in pregnancy (case studies)
5. Distribution of LLINs
6. Counseling PWs on IPTp and LLINs
7. Record keeping and reporting
8. Logistics and supply chain management
9. Monitoring and support supervision

Methodology

Several participatory methods were used considering the training objectives and nature of participants (adult/trainers). Almost every participant had hands - on experience during the training supported by the national trainers. The methods that were commonly used included the following:

- Min -Lectures for introduction and background information especially factual data.

- Group discussion and small group works
- Brainstorming
- Demonstrations
- Role plays
- Case studies and
- Question and answer sessions

Outcomes

a) Achievements

- The training was successful in that all the 24 expected participants (100%) attended for the three full days.
- Facilitation was participatory, participants contributed by sharing their experiences, asked whenever there were challenges and the facilitators fully discussed and answered their questions/concerns.
- All sessions started on time each day everything was covered within the three days.
- Participants were able to identify their strengths and gaps during this training and were given constructive feed back to improve on them.
- Each participant was oriented on the use of the ANC job aid
- Knowledge gain assessment through the pre and post test showed that the participants gained knowledge with the highest score as 96% and lowest as 48% in the pre-test while the highest was 100%(8 participants scored 100). The best improved did so by 52% (Annex 1)

b) Lessons learnt

1) When parteners ' support to the District stopped, some Health Facilities have run out of important HMIS tools like the Integrated ANC registers .
2) Most Health Workers still define severe Malaria using support parameters like hyper-parasiteamia.
3) In treatment of severe malaria, they still use Intra-venous Quinine especially for mothers in the 1st trimester and the practice by most health workers is to give salbutamol with each quinine dose.
4) Partner involvement in health promotional activities eases communication and promotes participation of the beneficiaries

5) Most participants felt that majority of the supervisors do sport checks than support supervision which compromises quality of service.

c) Challenges

a) Hands on practice on use of the gestation wheel may not be possible because the wheels are not available.
b) Late reporting by the participants on day one and delay to start the training
c) Some questions in the pre and post tests are very complicated and they are not very clear to participants.
d) Many participants came with maids who increased some of the costs for refreshments and meals.

d) Suggestions for improvement

I. The Health workers should be provided with gestation wheels, reviewed, updated materials (Mother- Baby pass ports, ANC register) and commodities to ease their work.
II. Supervisees should be supervised by people who have the same training background as them; they should be of a higher rank and with better experience and use a check list.
III. Some of the questions in the pre and post -test like (Q 8 in Pretest and 6 in Post-test, Q 5 in Pretest and 4 in Post test) should be revised so that participants understand them better.

e) Follow up action points and plan

1. The trainees/participants should organize and do a CME on Malaria in Pregnancy within 2 weeks after this training.
2. The district should ensure that all facilities have the integrated ANC registers.
3. MOH and Malaria Consortium should organize for immediate support supervision and then continue with regular technical support supervision.

Training report – Moyo

DATE: 25th to 27th MAY 2015

EXECUTIVE SUMMARY

The following report is about the activities conducted during the Malaria in Pregnancy training for Moyo District frontline health workers .It took place in Pent House Inn Hotel between 25thand 27th May 2015.

The training course was planned to last 3 days and it targeted health workers that provide care to pregnant women especially those that give ANC services.

A total of 24 (five males, nineteen females) participants were identified to take part in this training.

These included, (see table below):

Cadre	Female	Male	TOTAL
Nurses &Midwives	15	1	16
Nursing Assistants	5	1	6
Medical Officers	0	2	2
**TOTAL**	**20**	**4**	**24**

Background

The World Health Organisation (WHO) recommends intermittent preventive treatment (IPTp) with Sulfadoxine-Pyrimethamine (SP) for the prevention and control of malaria in pregnancy in all areas with moderate to high malaria transmission in Africa. The government of Uganda has set a target of 85 percent of pregnant women receiving at least two doses of IPTp (IPT2) delivered as part of the focused antenatal care (ANC) package. However, despite high ANC attendance and increased efforts to improve IPT2 coverage by government and implementation partners, this target is far from being met.

The ministry of health recognises some of the challenges associated with IPTp uptake Uganda such as; lack of knowledge about IPTp policy guidelines among health workers, poor data recording at health facility level and inadequate support supervision.

The purpose of this training therefore, was to update health workers knowledge about IPTp and improve the health management information system through training of frontline health service providers, particularly ANC staff.

The training emphasized the provision of improved ANC services as a way of increasing uptake of especially Malaria in Pregnancy prevention services.

Objectives

3. To improve health workers knowledge in management of malaria in pregnancy through updating them about the MIP policy
4. To orient Health Workers on records management including the job aid for MIP

Content

1) Malaria in pregnancy- effects on the mother and baby
2) Review of goal oriented ANC
3) National Malaria in Pregnancy policy and strategy
4) Management of malaria in pregnancy (case studies)
5) Distribution of LLINs
6) Counseling Pregnant Women on IPTp and LLINs
7) Record keeping and reporting
8) Logistics and supply chain management
9) Monitoring and support supervision

Methodology

The training was interactive and several methods of training and tools were used to ensure all participants benefitted from the rich knowledge source in the materials and from the trainers. Group discussions, role-plays, and demonstrations were used for this purpose (under the guidance of trainers).

A short meeting was held between the Central Trainers (MOH) and the District Trainers one day before the training.

This was intended to share updates made in the training materials with the district trainers and also to agree on terms of reference plus preparing all the needed materials and logistics.

The training was the officially opened by the Ag DHO Mr. Adruwa Micheal who was introduced by the malaria focal person Mr. Amoko Stephen. The DHO emphasized the importance of the training and that it had come at the right time. He was grateful to Malaria Consortium for the funding the activity and collaborating well with the District.

And through the methods below the training was conducted.

- Min-Lectures for introduction and background information especially factual data.
- Group discussion and small group works
- Brainstorming
- Demonstrations
- Role plays
- Case studies
- Question and answer sessions

Achievements

- Most of the participants' objectives were met and all sessions covered within the scheduled period. All sessions were presented to the trainees in a timely manner and as scheduled
- A total of twenty four (24) District frontline health workers were trained to offer standard ANC and other Malaria in pregnancy related services.
- Participants were able to identify their strengths and gaps during this training and constructive feedback was given to improve on them.
- Each participant was oriented on the use of the ANC job aid
- Through the Pre/post, we were able to assess for Knowledge gaps and gain before and after training respectively. An increase in knowledge level during training was demonstrated. As observed from the pre and post-test results, all participants gained new knowledge in the various areas of goal oriented ANC including prevention and management of Malaria in pregnancy.

Key Observations

a) All participants gained knowledge as is seen in the pre and post- test results.

b) All participants were available all the time and they participated actively

c) Most health workers have never seen/used a gestation wheel and therefore a knowledge gap was identified.

d) Most facilities do not have gestation wheels.

e) Facilitators observed that the training venue was convenient for the training.

f) Trainers were so much committed and had daily meetings to address arising issues.

g) Time management was excellent and the trainees were very interested.

Constraints

- Late reporting by the participants on day one and delay to start training.
- Continuity of the hands on practice on use of the gestation wheel is not possible because the wheels are not available so the acquire skill on use may be lost.
- Some category of cadres among the participants seemed to find it hard to understand and appreciate some aspects of the training.

Recommendations

1. All other health workers in the district that provide Malaria In pregnancy and other ANC services should be updated about the changes in IPTp policy.
2. Those that have been trained should plan for and conduct CMEs when they return to their facility so that the knowledge and skills acquired can be shared with other staff at the Health facility.
3. To sustain the quality of the knowledge and skills acquired during the training, all health workers that have been trained on Malaria in pregnancy should be followed up and supervised immediately and regularly.
4. The district in liaison with partners should identify a budget to procure and avail gestation wheels.
5. At the end of the training, with guidance from the trainers the trainees should generate an action plan per person/health facility on how they intend to use the acquired knowledge and skills for better service delivery.
6. These action plans should be used as reference points during support supervision.

Conclusion:

Provision of ANC services including those related to Malaria in Pregnancy is still challenged by the low uptake. This is mainly because of the poor quality of service in terms of prevention, treatment, and logistics and records management. Frontline health care providers for this kind of services need to be regularly updated as a way of addressing

arising gaps in knowledge and skills. But for sustainability and quality assurance, training should supported by prompt and regular technical support supervision.
