# Supplementary material for: Improving health worker performance through text messaging: A mixed-methods evaluation of a pilot intervention designed to increase coverage of intermittent preventive treatment of malaria in pregnancy in West Nile, Uganda
Source: PLoS One. 2018 Sep 6;13(9):e0203554. doi: 10.1371/journal.pone.0203554 (PMC6126848; doi:10.1371/journal.pone.0203554)
Supplement: S3 File — (PDF) [file pone.0203554.s007.pdf]

Focus group discussion guide – health workers

Participant Information

Participant code	Gender	Job title	Facility level	Public/PNFP

Obtaining consent

Before starting the FGD, informed written consent from the participant must be obtained. Please follow the script provided:

Good morning/ good afternoon

My name is [name] and this is my colleague [name]. We work for Malaria Consortium, a not-for-profit organisation that specialises in the prevention, control and treatment of malaria and other diseases. In collaboration with the Ministry of Health in Uganda, we are currently carrying out a study to explore ways of improving the delivery of care for e malaria in pregnancy (MIP). You have been invited to this discussion because your health facility has been involved in this study and we would like to hear your opinions on certain aspects of the study. Your names were suggested to us by the [DHO in your district/in-charge at your health facility].

Everything you say will be strictly confidential and your replies will only be shared with the other researchers working on this study. You will be identified by a number, not your name. This is why we have given each of you a badge with a number.

We have put together an information sheet which summarises the purpose of the discussion, why you've been selected and what happens if you agree to participate. Please take a moment to read through the information provided on the sheet.

[hand out participant information sheet and give time to read – sheet is kept by participants]

Do you have any questions about the information provided on this sheet?

Remember that you do not have to take part in the discussion if you prefer not to. You do not have to answer questions you don't feel comfortable with and can leave the discussion at any time.

If you agree to participate, we will audio-record the discussion and take notes. This will help us remember what you are telling us. We will destroy the audio-recording once the report about the discussion has been written.

Can I please ask you to also read this consent form?

[hand out consent form and give time to read]

Do you have any questions about the information in this form? If you consent to participating in the discussion, can I please ask you to sign the form?

[participants and researcher sign consent form, form is kept by researcher]

Thank you very much for agreeing to participate in the discussion. I will now switch on the audio recorder.

Discussion guide (intervention)

I would like to talk about the text messages you all received on malaria in pregnancy in July this year.

1. **Do you remember receiving these text messages? Can you tell me in your own words what they were about?**
   - How many messages did you receive?
   - Did you read every message/more than half/half/less than half/none?
   - If you did not read all of the messages, why not?
   - What were you typically doing when you received the text messages?
   - Would you usually read the text messages as soon as they arrived or did you read them at another time? If at another time, when?

2. **Did issues relating to your mobile phone ever mean that you did not receive the text messages as you should have - at 10 am every day Monday-Friday?**
   - Do you own one or more mobile phone? Do you own one or more SIM cards?
   - Roughly how many times per month is your phone out of battery for a day or longer?

- Do you often have problems with phone signal so that you are not able to send or receive text messages for a day or longer? Are you ever not able to receive text messages for other reasons?
- Is not having enough phone credit to receive text messages ever a problem for you?

3. What did you think of the messages? Did you like receiving them?

- Did you think they were all clearly worded and easy to understand? Would you have preferred the texts to have been in a different language to English?
- What did you typically do with the information you were given in the text messages?
- Who did you think was sending the text messages?
- Over the past few months have you received messages from the same number (xxxx) that were not about malaria in pregnancy? If so, what were they about? Roughly how many and how often do you receive them?
- Do you think you are getting too many messages from this number?
- Did you think the text messages added a lot to the work load you already had? Did you have time to read all of the texts?
- Would you been interested in receiving more text messages in the future on subjects other than malaria in pregnancy?

In June some of you attended a three-day training session on malaria in pregnancy. If you didn't attend the training, you may have heard about it from your colleagues.

4. Please can you put your hand up if you attended this training

- If you did not attend the training- what did your colleagues tell you about the training? What did they tell you about the new IPTp guidelines?
- If you did attend the training- Did you feel the new IPTp guidelines were clearly taught in this training? Please can you explain what the new IPTp guidelines said?

5. Did you think the text messages reminded you of what you had learnt during the malaria in pregnancy training?

- Did the texts clearly relate to what you had learnt in the training?
- Was any of the information in the text messages new to you?
- Did the text messages help you to understand anything you hadn't understood from the training?
- Did the text messages help you to remember anything you had forgotten from the training?
- Do you feel the text messages helped you to better understand the changes to the guidelines surrounding IPTp?

6. In your opinion are the text messages a good way to improve HW knowledge and understanding of training and guidelines?

- Did you feel the text messages helped you in your job? Why?
- Can you think of other methods you would have preferred?
- Can you suggest any ways the texts could have been made more appealing/fun/easier to read?

At the beginning of July a job aid referring to how to complete the ANC register was distributed to your health facility. *(Show the job aid)*

7. **Have you seen this job aid in your health facility?**
    - If you wanted to use the job aid to help you complete the ANC register was it always available to you?
    - How regularly do you think you used the job aid?

8. **Did you feel the job aid helped you in the completion of the ANC register?**
    - Was it clear how to correctly complete the ANC register when you looked at the job aid?
    - Do you think the job aid made you better at completing the ANC register?

9. **Can you suggest ways the job aid could been improved/made more useful?**
    - Would you have liked its appearance to change at all?
    - Would you have liked its format to change at all?

Discussion guide (control)

In June some of you may have attended a three day training session on Malaria in Pregnancy and if you didn't attend the training you may have heard about it from your colleagues.

1. **If you did not attend the training, what did your colleagues tell you about the training?**
    - What information did they tell you about the training?
    - Did you have a formal meeting about it or was it a more informal conversation while at work or at lunch etc?
    - What did your colleagues tell you about the IPTp guidelines?

2. **If you attended this training can you tell me what you learnt during the training?**
    - What did you think of the training?
    - What did you learn about IPTp guidelines?
    - Did you feel you understood IPTp at the end of the training?
    - If not, what are you still unclear about?
    - After the training would you have liked there to have been a follow on activity to help you remember the guidelines? Can you think of some ideas of what this could have been?

3. **If you could have chosen to receive text messages summarising the key MIP messages learnt during the training, one message every day for 5 weeks after the training, would you have wanted to do this?**
    - Do you think this would have helped you to remember better the key MIP messages?
    - If not, what doesn't appeal to you about this idea?
    - Have you ever had these sorts of messages sent to you to remind you of other training courses?

- If so please can you describe the content of the texts and how many you received?

At the beginning of July a job aid referring to how to complete the ANC register was distributed to your health facility. *(Show the job aid)*

4. **Have you seen this job aid before in your health facility?**
   - Where was the job aid usually kept in your HF? If you wanted to use the job aid to help you complete the ANC register was it always available to you?
   - Was the job aid usually used by just one health worker in your facility or was it shared among staff? If it was shared, were you ever not able to use it because somebody else was using it?
   - Can you think of other similar tools which are available in the HF where you work? Are these tools also to help you complete the ANC register or are they for other uses? If other uses, please describe them.
   - If you have other tools concerning the completion of the ANC register, does the information agree with the job aid or did this cause confusion?

5. **Did you like the appearance of the job aid?**
   - Did you like the format of the job aid, as a desk calendar? Or would you have preferred it to have been in a different format, is so what format?

6. **Did you feel the job aid helped you in the completion of the ANC register?**
   - Was it clear how the job aid related to the ANC register?
   - Was it clear how to correctly complete the ANC register when you looked at the job aid?
   - Were there any areas of the ANC register that you were unclear on how to complete and which were not covered in the job aid? If so, what were they?
   - Do you think the job aid made you better at completing the ANC register?

7. **How regularly do you think you used the job aid?**
   - once everyday/more than once everyday/once a week/less than once a week/only used it once/not at all?
   - If you did not use the job aid that much, why not?
   - Do you have suggestions of tools which may have been more useful for completing the ANC register than the job aid? If so, please describe them.

In-depth interview guide – district health officials

District Health Officers

Section A (intervention only)

1. **What are your thoughts on the MIP text messages sent out as part of this study?**
- Do you think the text messages compliment well the material taught during the MIP training?
- Do you think the text messages improve the effect of the training on the health worker's performance?

2. **Did you get any feedback from the health workers or biostatistician about what they thought of text messages and whether they liked receiving them?**
- Did health workers seem to appreciate receiving text messages?

3. **Do you think it is a reasonable amount of work to add to HWs work load to ask them to receive and read a text message every day over a set time period?**

4. **In your opinion would health workers continue to be interested in information sent to them in text messages and continue to cascade it?**

5. **Do you think the text messages are enough by themselves to improve health workers understanding of training or do you think other support is needed?**
- Can you suggest some support mechanisms which may be appropriate?

6. **Do you foresee any changes in the near future to how the mTrac system operates which would affect the use of this system to send out similar text messages?**

Section B (intervention and control)

Before health workers were sent these text messages some of them attended MIP training over 3 days, this training session covered all parts of the MIP guidelines and the updated IPTp recommendations. The training followed the Ministry of Health model where some, but not all, health workers from each HF are trained on the assumption that these HWs would then return to the HF and "cascade" the information to their colleagues who were not trained.

7. **In your opinion is this cascade approach a good one?**
- Do you believe the information is successfully cascaded to the HWs who do not attend the training?
- If not, what do you believe to be the main barriers to this cascading of information?
- What do you think are the strengths and weaknesses of this approach?
- Do you believe this approach is popular among health workers and those leading the training?

8. **For the future roll out of new policies and guidelines do you think the cascade approach plus text messages is a well suited one which should continue to be used?**

- What do you like and not like about the cascade approach to training?

It would be helpful for us to understand any relevant changes in the district which might have had an impact on malaria in the area, or ANC services or anything related.

9. **Can you think of any recent changes?**

10. **Do you know of any problems with the supply of ANC related drugs and equipment in the area recently?**

11. **Do you know of any problems with the supply of SP for IPTp?**

12. **In your opinion has there been any change in the levels of supervision provided in the health facilities during the time the study has been carried out? If so please can you describe the type and purpose of the increased supervision?**

Biostatistician

1. **What do you think of the texts that were sent to health workers on the subject of malaria in pregnancy?**

2. **Are texts perceived as a feasible way to improve health worker knowledge?**

3. **Is it a reasonable amount of work to add to your pre-existing work load?**

- Can you estimate how much of your time was taken up with the messages?
- Can you talk us through all of the steps that you take to send the messages out?
- Did you feel this was too much work or you were OK to manage it along with your other roles and responsibilities?
- I know for this study Malaria Consortium gave you some funds towards the internet bundle needed for sending the texts but if they had not, who would usually pay for this?

4. **Did you enjoy the role of managing the text messages**?

- Did the job of sending the text messages seem very different to you from your usual work or did you think the text messages complimented nicely the other uses of mTrac?

5. **Do you foresee any technical challenges in the use of the mTrac system for similar message sending over a longer period in the future?**

- If so please can you detail these challenges?

6. **Would a Biostatistician have the time and interest in the future to manage changes such as mobile phone numbers changing, people not wanting to receive texts any more, new people wanting to receive text messages?**

- If not you, then who do you suggest would be best equipped to do this?
- Do you think this is a role which can be incorporated in to an existing role or do you think it would require its own designated coordinator?

7. **What do you think of the sending of text messages as a way of complimenting class room training?**

- Work just as well for subjects other than MIP?
- Can you think of any ways the approach can be improved?
