# Supplementary material for: Improving health worker performance through text messaging: A mixed-methods evaluation of a pilot intervention designed to increase coverage of intermittent preventive treatment of malaria in pregnancy in West Nile, Uganda
Source: PLoS One. 2018 Sep 6;13(9):e0203554. doi: 10.1371/journal.pone.0203554 (PMC6126848; doi:10.1371/journal.pone.0203554)
Supplement: S4 File — (PDF) [file pone.0203554.s008.pdf]

Knowledge questionnaire

Section 1

**Gender:**	☐	Male	☐	Female
**Job title:**				
**Date:**				
**Are you involved in the provision of antenatal care services?**	☐	Yes	☐	No
**For how many years have you been involved in the provision of antenatal care services?**				

Section 2

Below are ten questions about malaria in pregnancy and IPTp. For each question, four answer options have been provided. Please tick all the answers you think are correct. Note that for some questions only one of the answer options is correct, whereas for other questions there may be more than one correct answer.

1. Who is more likely to get malaria – pregnant women or those who are not pregnant?

☐	Pregnant women are more likely to get malaria because their immunity is low.
☐	Women who are not pregnant are more likely to get malaria because they spend more time outside and are more likely to get bitten by mosquitoes.
☐	Pregnant women and those who are not pregnant are equally likely to get malaria.
☐	Pregnant women are more likely to get malaria because many pregnant women take folic acid which reduces their immunity to malaria.

2. What are possible effects of malaria infection during pregnancy on mother or baby?

☐	It can lead to severe anaemia in the mother.
☐	It can lead to spontaneous abortion, still births, premature labour, and low birth weight.
☐	It can affect the growth of the baby.
☐	It can mean the baby is born with some immunity to malaria

3. Is it difficult to determine if a pregnant woman is infected with malaria?

☐	Yes, because the malaria parasite may not be detectable in the woman's bloodstream.
☐	Yes, because pregnant women may be infected with the malaria parasite, but they may not show any symptoms of the disease.
☐	No, there is no difference in how easy it is to diagnose malaria between pregnant women and those who are not pregnant.
☐	No, because pregnant women are weaker. Whenever they are infected with malaria, they are very ill and so it is easy to diagnose.

4. What is the name of the drug used for intermittent preventive treatment for malaria in pregnancy (IPTp)?

☐	Quinine
☐	Sulfadoxine-pyrimethamine or SP
☐	Artemisinin-based combination therapy or ACT
☐	Doxycycline

5. When is it safe to give IPTp to a pregnant woman?

☐	It can be given safely at any time during a woman's pregnancy.
☐	It is safe from the start of the second trimester until the time of delivery.
☐	It can be given safely starting from the second trimester, but should not be given during the final stages of a woman's pregnancy.
☐	It can be given safely at any time in the first two trimesters of the pregnancy.

6. **How often should a pregnant woman receive IPTp?**

☐	Pregnant women should receive IPTp every time she attends antenatal care from the beginning of the second trimester, as long as doses are given at least one month apart.
☐	Pregnant women should only be given IPTp twice – the first dose between weeks 18 and 24 and the second dose between weeks 28 and 32.
☐	Pregnant women should be given IPTp three times: one dose at each antenatal care visit until the three doses have been completed.
☐	The number of doses depends on the pregnant woman and how she is feeling. It must be determined on an individual basis.

7. **A pregnant woman tells you she 'reacted' to IPTp in previous pregnancies. Which of the following statements are correct in this scenario?**

☐	The pregnant woman tells you she felt dizzy and nauseous after taking IPTp in the past. She should be given IPTp as those are mild and normal side effects.
☐	The pregnant woman tells you she felt dizzy and nauseous after taking IPTp in the past. She should not be given IPTp as this could mean she is allergic to sulphur-containing drugs.
☐	The pregnant woman tells you she experienced side effects such as a rash or difficulty breathing. She should not receive IPTp as this could mean she is allergic to the drug.
☐	Pregnant women who tell you they reacted to IPTp in the past should always be given IPTp as the benefits of IPTp outweigh any risks.

8. **Which pregnant women should not receive IPTp?**

☐	HIV positive pregnant women who are taking ART should not take IPTp.
☐	HIV positive pregnant women who are taking a drug co-trimoxazole or other sulpha-containing drugs should not be given IPTp.
☐	Pregnant women who have been treated for a case of malaria in the last four weeks should not receive IPTp.
☐	Pregnant women who have been feeling tired and weak on the day of the antenatal care visit should not receive IPTp.

9. **How should a health worker react if a woman requests to take the drug in her home rather than at the health facility, for example because she has not eaten?**

☐	Health workers should respect the woman's request and allow her to take the drug away from the facility.
☐	Health workers should only allow a pregnant woman to take the drug home if she is accompanied by someone who promises to ensure that she actually takes the drug, for example her husband.
☐	Health workers should encourage the woman to take the drug at the health facility under the supervision of the health workers.
☐	Health workers should tell pregnant women about why IPTp is important and that it is safe.

10. **What other advice with regard to malaria in pregnancy should a health worker give to a pregnant woman?**

☐	Health workers should tell the pregnant woman that IPTp protects her from malaria and so she will not get malaria in the future.
☐	Health workers should remind the pregnant woman to go to a health facility to be tested and treated if she feels ill or has symptoms of malaria in the future.
☐	Health workers should remind the pregnant woman she must continue to use her mosquito net.
☐	Health workers should tell the pregnant woman to come straight to the ANC if she has malaria symptoms in the future so she can be given another dose of IPTp.

Section 3
To be completed by researcher

**Facility code**		
**Health worker code**		
**District**	☐ Moyo	☐ Adjumani
**Facility level**	☐ HC II<br>☐ HC III	☐ HC IV<br>☐ Hospital
**Facility type**	☐ Public	☐ PNFP
